# EGCG Enhances the Therapeutic Potential of Gemcitabine and CP690550 by Inhibiting STAT3 Signaling Pathway in Human Pancreatic Cancer

**DOI:** 10.1371/journal.pone.0031067

**Published:** 2012-02-13

**Authors:** Su-Ni Tang, Junsheng Fu, Sharmila Shankar, Rakesh K. Srivastava

**Affiliations:** 1 Department of Pharmacology, Toxicology and Therapeutics, and Medicine, The University of Kansas Cancer Center, The University of Kansas Medical Center, Kansas City, Kansas, United States of America; 2 Department of Pathology and Laboratory Medicine, The University of Kansas Cancer Center, The University of Kansas Medical Center, Kansas City, Kansas, United States of America; Technische Universität München, Germany

## Abstract

**Background:**

Signal Transducer and Activator of Transcription 3 (STAT3) is an oncogene, which promotes cell survival, proliferation, motility and progression in cancer cells. Targeting STAT3 signaling may lead to the development of novel therapeutic approaches for human cancers. Here, we examined the effects of epigallocathechin gallate (EGCG) on STAT3 signaling in pancreatic cancer cells, and assessed the therapeutic potential of EGCG with gemcitabine or JAK3 inhibitor CP690550 (Tasocitinib) for the treatment and/or prevention of pancreatic cancer.

**Methodology/Principal Findings:**

Cell viability and apoptosis were measured by XTT assay and TUNEL staining, respectively. Gene and protein expressions were measured by qRT-PCR and Western blot analysis, respectively. The results revealed that EGCG inhibited the expression of phospho and total JAK3 and STAT3, STAT3 transcription and activation, and the expression of STAT3-regulated genes, resulting in the inhibition of cell motility, migration and invasion, and the induction of caspase-3 and PARP cleavage. The inhibition of STAT3 enhanced the inhibitory effects of EGCG on cell motility and viability. Additionally, gemcitabine and CP690550 alone inhibited STAT3 target genes and synergized with EGCG to inhibit cell viability and induce apoptosis in pancreatic cancer cells.

**Conclusions/Significance:**

Overall, these results suggest that EGCG suppresses the growth, invasion and migration of pancreatic cancer cells, and induces apoptosis by interfering with the STAT3 signaling pathway. Moreover, EGCG further enhanced the therapeutic potential of gemcitabine and CP690550 against pancreatic cancer.

## Introduction

Signal transduction and activators of transcription (STAT) proteins is a family of cytoplasmic transcription factors which are initially present in inactive forms [1], [2]. They are stimulated by the binding of signaling peptides, such as cytokine, growth factors, and hormone, which results in dimerization of their cognate receptors and activation of tyrosine kinases such as Janus kinase (JAK). The activated tyrosine kinases could subsequently phosphorylate the cytoplasmic domains of receptors to provide recognition sites for non-phosphorylated STATs monomers. Once STATs are phosphorylated by activated tyrosine kinases after binding, they form homo or hetero-dimers via their Src-homology 2 (SH2) domain and rapidly migrate into the nucleus, where the dimers bind to DNA sequences to active specific gene transcription [1], [2].

Numerous experiments have demonstrated that normal physical functions of STATs are critical in regulating many aspects of cellular proliferation, differentiation, migration, and survival. Among all the STAT family members, STAT3 is the most intimately linked to cell survival and proliferation and tumorigenesis [3], [4]. It is widely expressed in most tissues and is considered as a potential oncogene. STAT3 is often constitutively active in many human cancer cells, including multiple myeloma, glioblastoma, leukemia, lymphoma, breast cancer, prostate cancer, lung cancer, and neck cancer [5], [6], [7]. STAT3 can be activated by multiple cytokines, including IL-6, IL-11, ciliary neurotrophic factor, and leukemia inhibitory factor, which all use gp130-type receptors. Interestingly, STAT3 can contribute to either apoptosis or survival in different organs and cell types. It can promote the proliferation in hepatocytes [8], neuron cells [9], and T cells [10], but is indispensable for the apoptosis in mammary [11] and myeloid cells [12].

STAT3 is a latent transcription factor that resides in the cytoplasm. Upon activation by tyrosine phosphorylation, STAT3 dimerizes, translocates to the nucleus and binds to nuclear DNA to modulate transcription of target genes. STAT3 phosphorylation is principally mediated through the activation of non-receptor protein tyrosine kinase family of JAKs, which include many members JAK1, JAK2, JAK3 and tyrosine kinase 2 [13], [14]. Additionally, the STAT3 phosphorylation can also be mediated by crosstalk with c-Src kinase [13], [14], [15]. The major phosphorylation sites in STAT3 include tyrosine and serine residues at positions Tyr^705^ and Ser^727^, respectively, located in the transactivation domain. The activation of STAT3 results in expression of many target genes required for tumor cell survival (e.g. Bcl-X_L_, Mcl-1 and survivin), proliferation (e.g. cyclin D1 and c-myc) and angiogenesis [e.g. vascular endothelial growth factor (VEGF)] as well as metastasis [16]. Thus, STAT3-signaling pathway has been a favorite therapeutic target for drug development [17], [18].

Gemcitabine (a nucleoside analog) showed more clinical benefit on pancreatic cancer patients compared with the conventional medications [19]. Some potent and selective JAK3 inhibitors, e.g. CP690550, demonstrated significant clinical activity in cancer [20], [21]. CP690550 represents only a starting point in the search for a safer small molecule immunosuppressant, and that an isozyme-selective JAK3 inhibitor identified by rational drug design might be substantially safer. In recent years, many new insights have been gained into the investigation on a variety of purified compounds from natural products. For instance, EGCG is the major catechin from green tea and has been recognized as an important chemopreventive agent and as modulators of tumor cell response to chemotherapy [22], [23], [24]. It has been shown to inhibit cell proliferation [25], induce apoptosis [26] in tumor cells, prevent angiogenesis [27], modulate the invasion and migration of cancers, and interfere with multiple signaling pathways, including the nuclear factor-κB signaling pathway [28], epidermal growth factor-mediated pathway [29], insulin-like growth factor-I signaling pathway [30], mitogen-activated protein kinase-dependent pathway [31], and proteasome degradation pathway [32].

In this paper, we examined the effects of EGCG on STAT3 signaling in human pancreatic cancer cells, and also assessed the interactive effects of EGCG with gemcitabine or JAK3 inhibitor CP690550 on their therapeutic potential. We found that EGCG inhibited the expression of JAK3 and STAT3 (phospho and total), STAT transcription and activation, and the expression of STAT3-regulated genes, resulting in the inhibition of cell motility, migration and invasion, and the induction of caspase-3 and PARP cleavages. Inhibition of STAT3 by shRNA in pancreatic cancer cells enhances the inhibitory effects of EGCG on cell migration and motility. Our results demonstrate that activation of the STAT3 signaling pathway is critical for the growth of pancreatic cancer cells and suggest that EGCG targeting STAT3 signaling may be a potential therapeutic intervention for pancreatic cancer. Furthermore, the combination of EGCG with gemcitabine or CP690550 had additive/synergistic effects on cell viability and apoptosis.

## Materials and Methods

### Cell lines and culture conditions

Human pancreatic cancer cell lines AsPC-1 and PANC-1 were purchased from the American Type Culture Collection (Manassas, VA), and cultured in RPMI 1640 medium supplemented with 10% fetal bovine serum (Thermo Scientific) and 1% antibiotic-antimycotic (Invitrogen) at 37°C in a humidified atmosphere of 95% air and 5% CO_2_.

### Cell transfection

STAT3 shRNAs were designed using BLOCK-iT™ RNAi Designer (Invitrogen).The accession number was obtained from the Gene bank. The sequences of STAT3 shRNAs (accession number: NM_139276) are corresponding to the coding regions 398–416 (5′- CCA CTT TGG TGT TTC ATA A-3′), 1070–1088 (5′-CCCGTCAACAAATTAAGAA-3′), 1448–1466 (5′-GCC TCT CTG CAG AAT TCA A-3′) and 1935–1953 (5′-GGA CAA TAT CAT TGA CCT T-3′) nucleotides. AsPC-1 and PANC-1 cells were transfected with a mixture of shRNAs using Lipofetamine 2000 (Invitrogen). After 24 h of transfection, cells were treated with EGCG. Cells were used for cell viability detection, scratch assay, qRT-PCR and western blotting.

### XTT Assay

Cells (1×10^4^ in 200 µl culture medium per well) were seeded in 96-well plate (flat bottom), treated with or without drugs and incubated for various time points at 37°C and 5% CO_2_. Before the end of the experiment, 50 µl XTT labeling mixture (final concentration, 125 µM XTT (sodium 2,3-Bis(2-methoxy-4-nitro-5-sulfophenyl)-2H-tetrazolium-5-carboxanilide inner salt) and 25 µM PMS (phenazine methosulphate) per well was added and plates were incubated for further 4 h at 37°C and 5% CO_2_. The spectrophotometric absorbance of the sample was measured using a microtitre plate (ELISA) reader. The wavelength to measure absorbance of the formazon product was 450 nm, and the reference wavelength was 650 nm.

### Caspase-3/7 Assay

Cells (3×10^4^ per well) were seeded in a 96-well plate with 200 µl culture medium. Approximately 16 h later, cells were treated with various doses of EGCG. Casapse-3/7 activity was measured as per manufacturer's instructions (Invitrogen).

### Scratch assay

AsPC-1 scrambled and STAT3 shRNA cells were seeded in 6 well dishes. When all the cultures were 50% confluent, a cross was marked in the center of each dish using a 10 µl tip. The cells were washed with PBS and cultured in fresh medium. The scratch pictures were taken under a fluorescence microscope at 0, 24 and 48 h for the same positions after the cells were treated with EGCG.

### Transwell migration assay

To determine the effect of EGCG on cell migration, AsPC-1 and PANC-1 cells were plated in the top of chamber onto the noncoated membrane (24-well insert; pore size, 8 µm; Corning Costar) at a density of 1×10^4^ cells/well in RPMI medium containing 1% FBS, and allowed to migrate toward RPMI medium containing 10% FBS in the lower chamber. EGCG was added to the both chambers to achieve the concentration of 0, 20, 40, 60 µM, respectively. After 24 h of incubation, cells were fixed with 4% paraformaldehyde and stained with crystal violet. The migrated cells were counted under a light microscope (four random fields per well).

### Transwell invasion assay

To determine the effect of EGCG on cell invasion, AsPC-1 and PANC-1 cells were plated in the top of chamber onto the Matrigel coated membrane (24-well insert; pore size, 8 µm; Corning Costar) at a density of 1×10^4^ cells/well in RPMI medium containing 1% FBS, and allowed to invade toward RPMI medium containing 10% FBS in the lower chamber. EGCG was added to the both chambers to achieve the concentration of 0, 20, 40, 60 µM, respectively. After 48 h of incubation non-invaded cells were removed by cotton swab, and invaded cells were fixed with 4% paraformaldehyde and stained with crystal violet. The invaded cells were counted under a light microscope (four random fields per well).

### Transient transfection and STAT3 reporter

AsPC-1 and PANC-1 cells were cultured in 100 mm dishes and transfected at 70% confluent with pGreenfire1-STAT3 reporter plasmid using Lipofetamine 2000 (Invitrogen). After 24 h, cells were treated with EGCG (0–80 µM). After incubation of 24 h, luciferase activity was determined using the Dual-Luciferase Reporter Assay System (Promega), according the manufacturer's instructions on a multilabel plate reader (Wallac Victor, Perkin-Elmer).

### RNA isolation and real-time RT-PCR

Total RNA was isolated from AsPC-1 and PANC-1 cells using TRIzol reagent (Invitrogen). RNA concentration was determined using Nano Drop 2000 Spectrophotometer. cDNA was synthesized and RT-PCR reactions were performed using SuperScript II (Invitrogen) according to the manufacturer's instructions. Real-time PCR was performed on the Applied Biosystems 7300 Real-time PCR System, using the following program: 50°C for 2 min, 95°C for 10 min, and then 40 cycles of 95°C for 15 s and 60°C for 1 min. PCR primers were purchased from Realtimeprimers.com. All reactions were performed in triplicate, and the relative expression of target mRNA in each sample was normalized with that of mean GAPDH.

PCR primers sequences: GAPDH, forward primer 5′- GAG TCA ACG GAT TTG GTC GT -3′; reverse primer 5′- TTG ATT TTG GAG GGA TCT CG -3′; STAT3, forward primer 5′- CCT TTG ACA TGG AGT TGA CC -3′; reverse primer 5′- TAA AAG TGC CCA GAT TGC TC -3′; Cyclin D1, forward primer 5′- TTC AAA TGT GTG CAG AAG GA -3′, reverse primer 5′- GGG ATG GTC TCC TTC ATC TT -3′; c-Myc, forward primer 5′- CGA CGA GAC CTT CAT CAA AA -3′, reverse primer 5′- TGC TGT CGT TGA GAG GGT AG -3′; Survivin, forward primer 5′- TCC CTG GCT CCT CTA CTG TT -3′, reverse primer 5′- TGT CTC CTC ATC CAC CTG AA -3′; VEGF, forward primer 5′- AGA CAC ACC CAC CCA CAT AC -3′, reverse primer 5′- TGC CAG AGT CTC TCA TCT CC -3′; BclX_L_ forward primer 5′- GCT CTC ACT CCC AGT CCA AA -3′, reverse primer 5′-GCT GAG GCC ATA AAC AGC TC -3′.

### Immunofluorescent staining

AsPC-1 and PANC-1 cells were cultured in RPMI medium containing 10% FBS and treated with EGCG (0, 40, 60 µM) for 24 h. Cells were then fixed with 4% paraformaldehyde and stained with antibodies against STAT3 (mouse monoclonal IgG1; Cell Signaling) at 4°C overnight. Cells were washed and again incubated with anti-mouse-FITC secondary antibody (Sigma) along with DAPI (0.5 µg/ml). Stained slides were mounted with mounting medium and visualized under a fluorescence microscope. For better visuality, the color of DAPI was changed from blue to red. The green color represents the expression of STAT3.

### Western blotting analysis

To detect different proteins, AsPC-1 and PANC-1 cells treated with EGCG (0–60 µM) were washed with PBS and lysed in RIPA buffer containing 1× protease inhibitor cocktail. The lysates were centrifuged and the supernatant was collected. Protein concentrations were determined using the Bio-Rad Protein Assay (Bio-Rad). Protein extracts (40 µg) were separated on 12.5% SDS-PAGE. Transferred membranes were blocked using 5% nonfat dry milk and incubated overnight with primary antibodies at 1∶1,000 dilutions in TBS, followed by secondary antibodies conjugated with horseradish peroxidase at 1∶5,000 dilutions in TBS-Tween 20 for 1 hour at room temperature. Membranes were developed using ECL Substrate. Protein bands were visualized on X-ray film using an enhanced chemiluminescence system.

### Statistical analysis

The mean and SD were calculated for each experimental group. Differences between groups were analyzed by one or two way ANOVA using PRISM statistical analysis software (GrafPad Software, Inc., San Diego, CA). Significant differences among groups were calculated at P<0.05.

## Results

### EGCG inhibits migration and invasion of pancreatic cancer cells, and induces caspase3 activity

**Figure 1:**
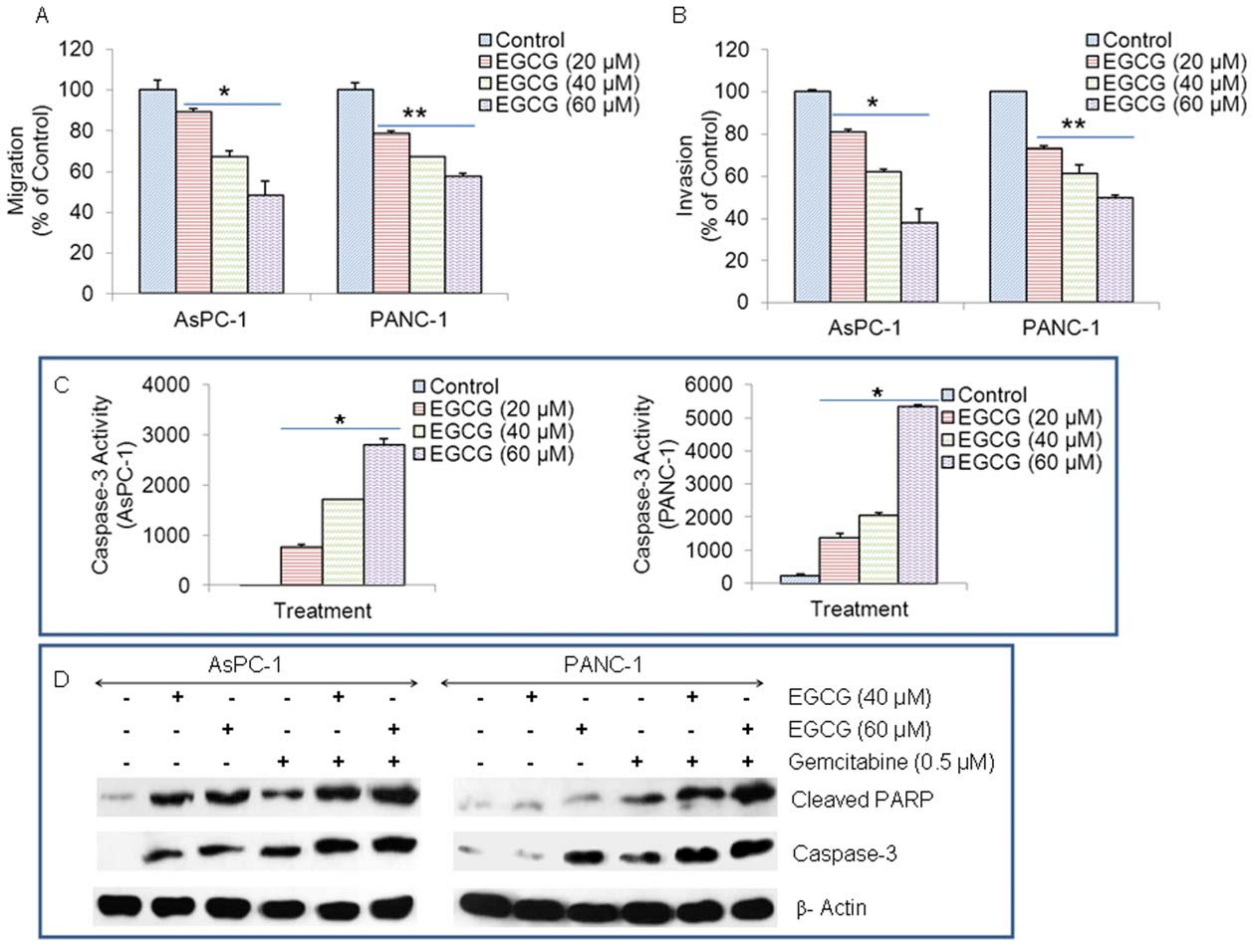
Effects of EGCG on pancreatic cancer cells. (A), Transwell migration assay. AsPC-1 and PANC-1 cells were plated in the top chamber of the transwell and treated with EGCG (0–60 µM) for 24 h. Cells migrated to the lower chambered were fixed with methanol, stained with crystal violet and counted. Data represent mean ± SD. * or ** = significantly different from respective controls, P<0.05. (B) Matrigel invasion assay. AsPC-1 and PANC-1 cells were plated onto the Matrigel-coated membrane in the top chamber of the transwell and treated with EGCG (0–60 µM) for 48 h. Cells invaded to the lower chamber were fixed with methanol, stained with crystal violet and counted. Data represent mean ± SD. * or ** = significantly different from respective controls, P<0.05. (C), Caspase-3 activity. AsPC-1 and PANC-1 cells were treated with EGCG (0–40 µM) for 48 h, and the caspase-3 activity was measured as per manufacturer's instructions (Invitrogen). Data represent mean ± SD. * = significantly different from respective controls, P<0.05. (D), AsPC-1 and PANC-1 cells were treated with EGCG (0–60 µM) with or without gemcitabine (0.5 µM) for 48 h. Cells were harvested and the Western blot analysis was performed to examine the expression of PARP and caspase-3. β-actin was used as a loading control. PARP antibody recognizes cleaved PARP, and caspase-3 antibody recognizes cleaved/active caspase-3.

It has been demonstrated that STAT3 plays an important role in regulating cell movement by controlling cytoskeleton reorganization and cell adhesion properties [33]. Due to the correlation between STAT3 and cell movement, we examined the effects of EGCG on the migration and invasion of AsPC-1 and PANC-1. Cells were plated in the top of chamber onto the noncoated membrane and the Matrigel coated membrane for migration and invasion detection, respectively. After the treatments of EGCG, the migrated and invaded cells were stained and counted. The results show that the migrated and invaded pancreatic cells reduced in a dose-dependent manner (Fig. 1A and B). These data suggested that EGCG can inhibit the migration and invasion of pancreatic cancer cells.

Caspase-3 is a member of the cysteine-aspartic acid protease (caspase) family and activated in the apoptotic cell both by extrinsic (death ligand) and intrinsic (mitochondrial) pathways [34]. EGCG induced caspase-3 activity in a dose dependent manner in both AsPC-1 and PANC-1 cells, as measured by fluorometric assay (Fig. 1C). These data suggest that EGCG can induce apoptosis by activating caspase-3.

### EGCG enhances gemcitabine-induced cleavage of caspase3 and PARP in pancreatic cancer cells

PARP is normally involved in DNA repair, DNA stability, and other cellular events, and cleaved by members of the caspase family during early apoptosis; therefore, it is a substrate for caspase activity and a reliable marker of apoptosis [35]. We next examined whether EGCG and gemcitabine interact together to cleave caspase-3 and PARP in AsPC-1 and PANC-1 cells. EGCG and gemcitabine alone induced cleavage of caspase-3 in both the cell lines. Furthermore, the combination of EGCG with gemcitabine induced significantly more caspase-3 cleavage than single agent alone (Fig. 1D). EGCG and gemcitabine alone showed PARP cleavage in AsPC-1 and PANC-1 cells. By comparison, the combination of EGCG with gemcitabine resulted in an enhanced PARP cleavage. These data suggest that EGCG can induce apoptosis by caspase-3 activation and PARP cleavage.

### EGCG inhibits JAK3/STAT3 pathway in pancreatic cancer

**Figure 2:**
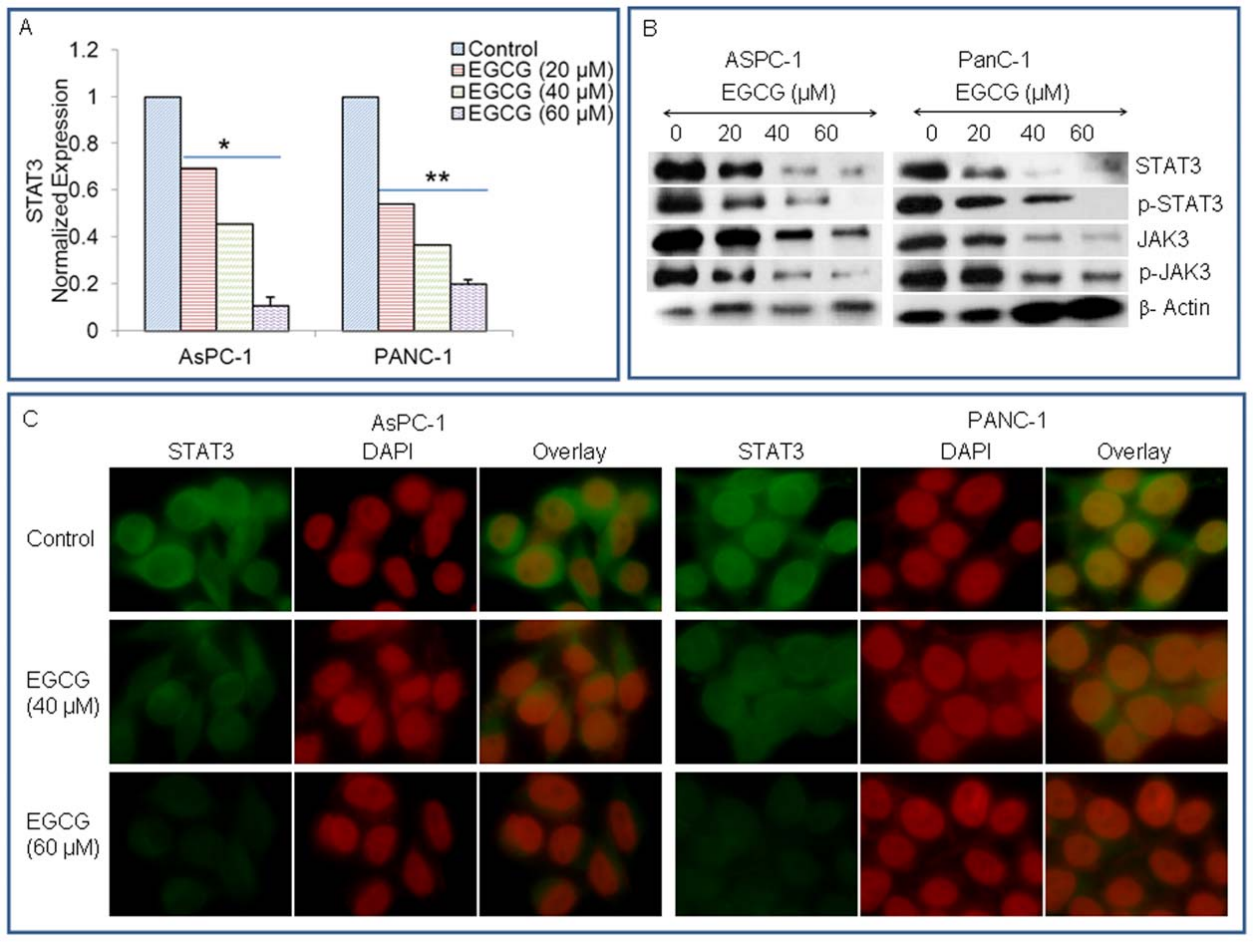
EGCG inhibits JAK3/STAT3 pathway in pancreatic cancer. (A), AsPC-1 and PANC-1 cells were treated with EGCG (0–60 µM) for 48 h, and the expression of STAT3 was measured by q-RT-PCR. Data represent mean ± SD. * or ** = significantly different from respective controls, P<0.05. (B), AsPC-1 and PANC-1 cells were treated with EGCG (0–60 µM) for 48 h. The expression of STAT3, p-STAT3, JAK3 and p-JAK3 was measured by Western blot analysis. β-actin was used as a loading control. (C), Expression of STAT3 in AsPC-1 and PANC-1 cells. Cells were treated with EGCG (0–60 µM) for 48 h. After incubation, the expression of STAT3 was measured by immunoflurescence. DAPI was used to stain nuclei. For better visuality, the color of DAPI was changed from blue to red. The green color represents the expression of STAT3. Red color = nuclei.

We next examined the effects of EGCG on expression of STAT3, phosphorylation of STAT3 and JAK3, and nuclear expression of phospho-STAT3 in AsPC-1 and PANC-1 cells. To examine the effects of EGCG on STAT3 expression, we performed qRT-PCR analysis (Fig. 2A).

EGCG inhibited the expression of STAT3 mRNA in both AsPC-1 and PANC-1 cells. We next measured the expression of total and phosphorylated JAK3 and STAT3 by the Western blot analysis (Fig. 2B). EGCG inhibited the phosphorylation of both JAK3 and STAT3 in a dose-dependent manner in AsPC-1 and PANC-1 cells. Surprisingly, the expression of both JAK3 and STAT3 was also inhibited by EGCG. These data suggest that EGCG can inhibit the expression of JAK3 and STAT3, as well as their post-translation modification.

Since STAT3 is constitutively active in pancreatic cancer cells, we next examined the effects EGCG on STAT3 expression by immunofluorescence. As shown in Fig. 2C, EGCG inhibited the expression of STAT3 (presence of the green color) in a dose-dependent manner in both the cell lines. These data suggest that inhibition of apoptosis by EGCG is associated with suppression of JAK3/STAT3 pathway.

### EGCG inhibits STAT3 transcription and expression of STAT3-regulated genes

**Figure 3:**
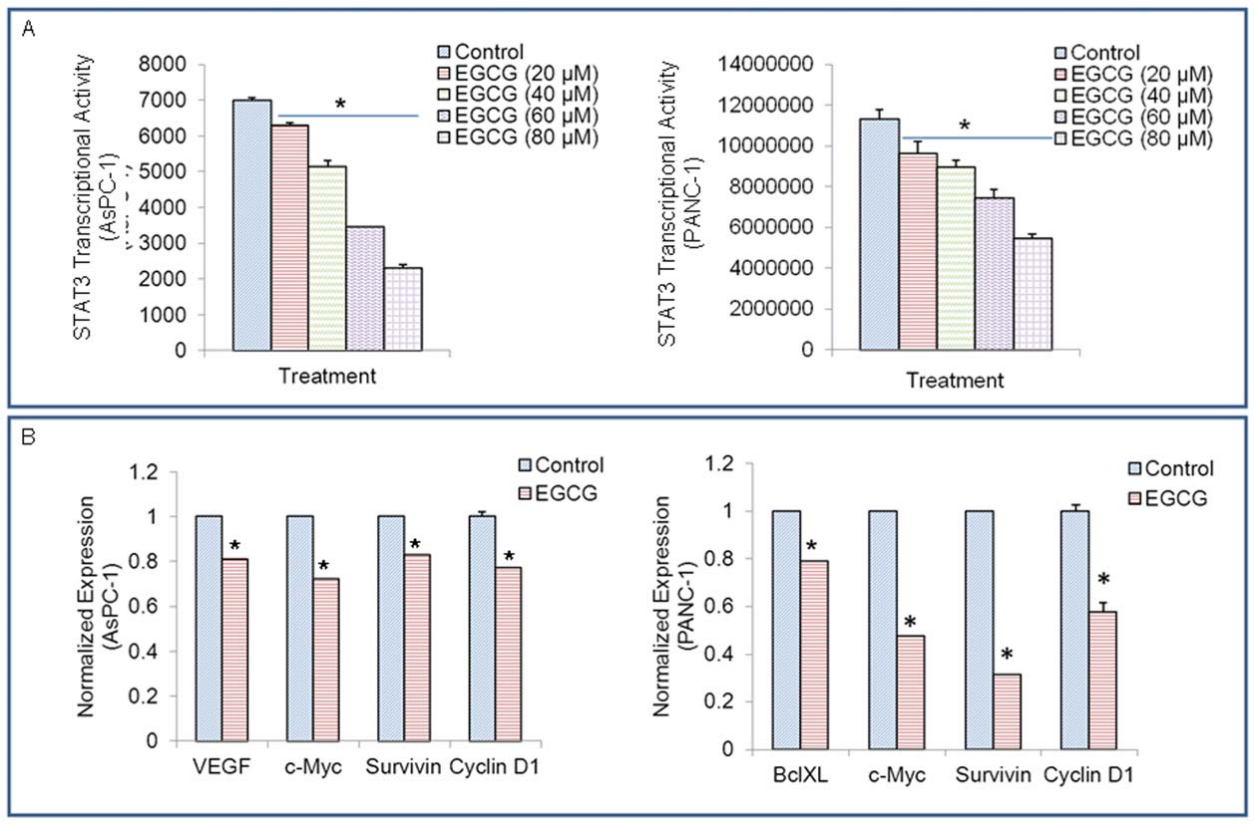
EGCG inhibits the expression of STAT3-regulated genes. (A), STAT3 activity. AsPC-1 and PANC-1 cells were transfected with pGreenfire1-STAT3 reporter plasmid. Cells were treated with EGCG (0–80 µM). After incubation of 24 hours, luciferase activity was determined using the Dual-Luciferase Reporter Assay System, according the manufacturer's instructions on a multilabel plate reader. Data represent mean ± SD. * = significantly different from respective controls, P<0.05. (B), VEGF, Bcl-X_L_, c-Myc, Survivin and Cyclin D1 were detected by qRT-PCR. Data represent mean ± SD. * = significantly different from respective controls, P<0.05.

STAT3 is considered as an oncogene because it is correlated with tumorigenesis [7]. Therefore, it is necessary to determine the effect of EGCG on STAT transcriptional activity. Pancreatic cancer cells were transfected with pGreen fire1-STAT3 reporter plasmid and treated with EGCG (0–80 µM). After incubation of 24 h, luciferase activity was determined by reporter assay. As shown in Fig. 3A, EGCG inhibited STAT3 transcriptional activity in a dose-dependent manner in pancreatic cancer AsPC-1 and PANC-1 cells.

Previous studies have shown that STAT3 can regulate the expression of many gene products involved in proliferation, cell survival, angiogenesis and anti-apoptosis [7]. For instance, VEGF, Bcl-X_L_, c-Myc, Survivin and Cyclin D1 are regulated by STAT3 activation [17], [36]. As illustrated in Fig. 3B, STAT3-regulated genes, c-Myc, Survivin and Cyclin D1 were inhibited by EGCG in AsPC-1 and PANC-1 cells. EGCG also decreased the expression of VEGF in AsPC-1 cells, and Bcl-X_L_ in PANC-1 cells. These data demonstrate that EGCG can inhibit the expression of STAT3-regulated genes in pancreatic cancer cells. These STAT-3 target genes have been shown to regulate cell proliferation, cell cycle, apoptosis and angiogenesis.

### Inhibition of STAT3 enhances the inhibitory effects of EGCG on cell motility and viability in pancreatic cancer cells

**Figure 4:**
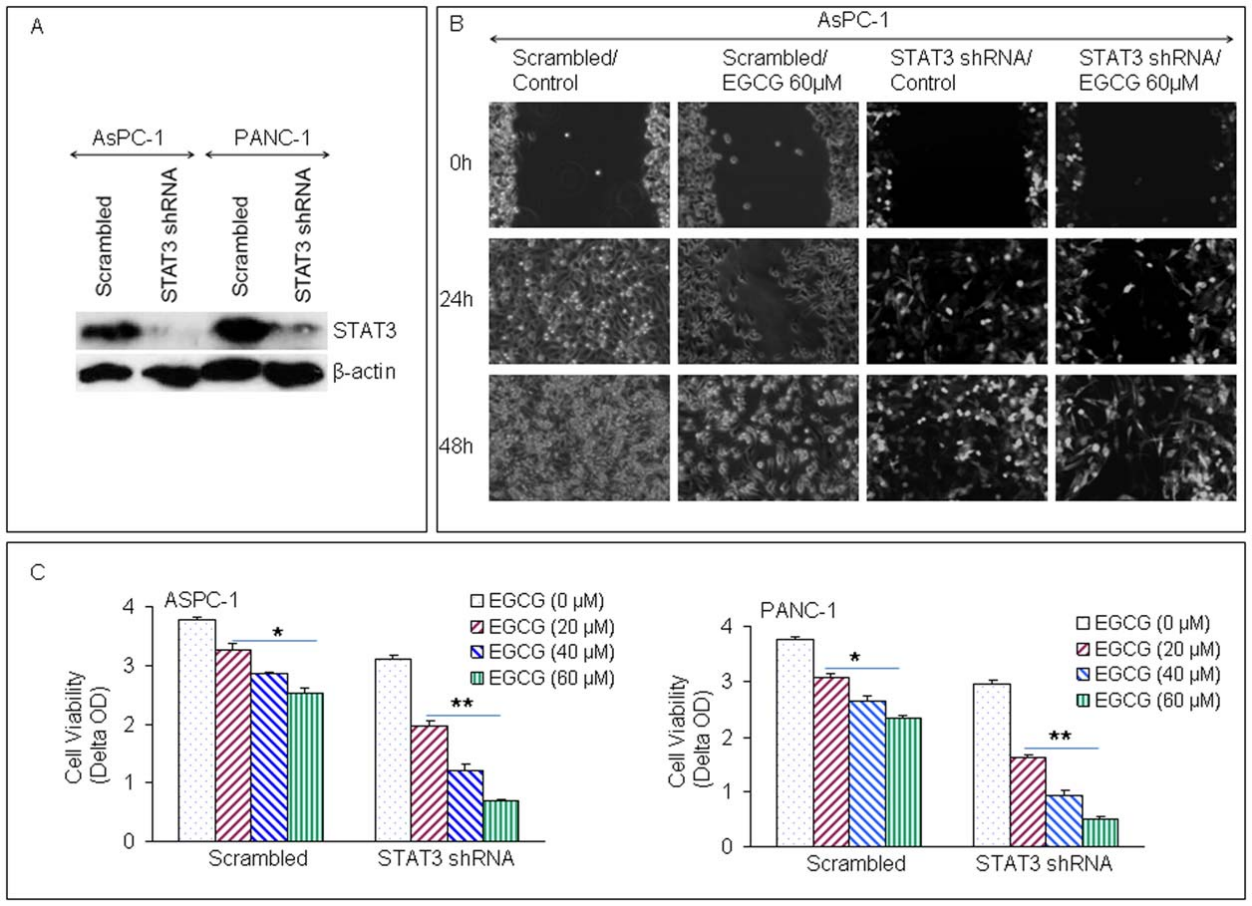
Inhibition of STAT3 enhances the inhibitory effects of EGCG on motility and cell viability of pancreatic cancer cells. (A), AsPC-1 and PANC-1 were transfected with STAT3 shRNA. The expression of STAT3 was performed by Western blotting. (B), AsPC-1 scratch assay. AsPC-1 scrambled and STAT3 shRNA cells were cultured in 6 well dishes. The scratch was marked when the dishes were 50% confluent. Pictures were taken after the cells were treated with EGCG and incubated for 0, 24 and 48 h. (C), Cell viability assay. AsPC-1 and PANC-1 (scrambled and STAT3 shRNA) cells were seeded and treated with EGCG (0, 20, 40, 60 µM). After 72 h of treatment, cell viability was performed by XTT assay. Data represent mean ± SD. * or ** = significantly different from respective controls, P<0.05.

In this work, STAT3 shRNA was used to silence STAT3 gene expression in AsPC-1 and PANC-1 cells. STAT3 shRNA inhibited the expression of STAT3 in both AsPC-1 and PANC-1 cells as demonstrated by the Western blot analysis (Fig. 4A). To examine the effects of EGCG on pancreatic cancer cells, scratch and cell viability assays were performed using both scrambled and STAT3 shRNA cells. In scratch assay, the inhibitory effects of EGCG on the migration of AsPC-1 cells were enhanced by inhibiting STAT3 gene expression (Fig. 4B). EGCG and STAT3 shRNA alone reduced percent of viable AsPC-1 and PANC-1 cells in a dose-dependent manner (Fig. 4C). Interestingly, the inhibitory effects of EGCG on cell viability were further enhanced by STAT3 shRNA in both the cell lines.

**Figure 5:**
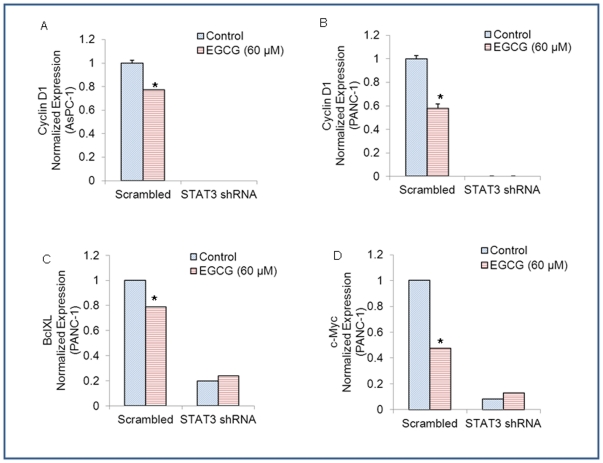
Effects of STAT3 shRNA on the regulation of cyclin D1, Bcl-X_L_ and c-Myc by EGCG. (A), AsPC-1/scrambled and AsPC-1/STAT3 shRNA cells were treated with or without EGCG (60 µM) for 48 h. The expression of cyclin D1 was measured by q-RT-PCR. Data represent mean ± SD. * = significantly different from respective controls, P<0.05. (B), PANC-1/scrambled and PANC-1/STAT3 shRNA cells were treated with or without EGCG (60 µM) for 48 h. The expression of cyclin D1 was measured by q-RT-PCR. Data represent mean ± SD. * = significantly different from respective controls, P<0.05. (C), PANC-1/scrambled and PANC 1/STAT3 shRNA cells were treated with or without EGCG (60 µM) for 48 h. The expression of Bcl-X_L_ was measured by qRT-PCR. Data represent mean ± SD. * = significantly different from respective controls, P<0.05. (D), PANC-1/scrambled and PANC-1/STAT3 shRNA cells were treated with or without EGCG (60 µM) for 48 h. The expression of c-Myc was measured by q-RT-PCR. Data represent mean ± SD. * = significantly different from respective controls, P<0.05.

We next examined the effects of STAT3 shRNA on the regulation of STAT3-target genes by EGCG. EGCG inhibited the expression of STAT3-regulated gene Cyclin D1 in both AsPC-1/scrambled and PANC-1/scrambled cells (Fig. 5 and B). STAT3 shRNA completely inhibited the expression of cyclin D1 in both pancreatic cancer cell lines in the presence or absence of EGCG. EGCG also inhibited he expression of Bcl-X_L_ and c-Myc in PANC-1/scrambled cells. Bcl-X_L_ and c-Myc were also inhibited in PANC-1/STAT3 shRNA cells compared to PANC-1/Scrambled cells. However, EGCG was unable to further inhibit the expression of Bcl-X_L_ and c-Myc in PANC-1/STAT3 shRNA cells. These data suggest that EGCG can regulate pancreatic cancer cell motility and viability which are associated with STAT3 pathway.

### Gemcitabine synergizes with EGCG to inhibit cell viability and induce apoptosis in pancreatic cancer cells

**Figure 6:**
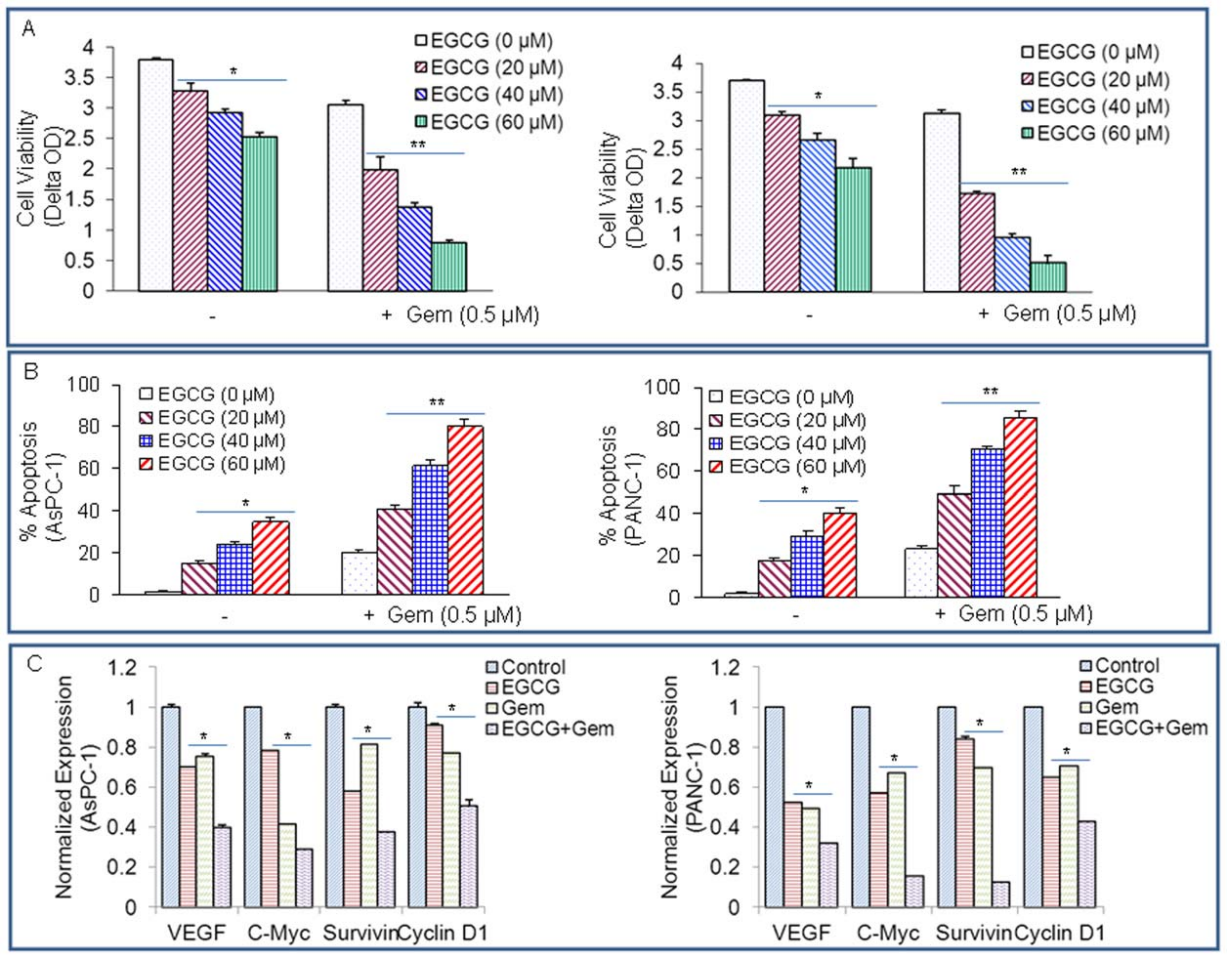
EGCG and gemcitabine inhibit cell viability and STAT3 target genes. (A), AsPC-1 and PANC-1 cells were treated with EGCG (0, 20, 40, 60 µM) with or without gemcitabine (0.5 µM) for 72 h. Cell viability was measured by XTT assay. Data represent mean ± SD. * or ** = significantly different from respective controls, P<0.05. (B), AsPC-1 and PANC-1 cells were treated with EGCG (0, 20, 40, 60 µM) with or without gemcitabine (0.5 µM) for 72 h. Apoptosis was measured by TUNEL assay. Data represent mean ± SD. * or ** = significantly different from respective controls, P<0.05. (C), Inhibition of STAT3 target genes by EGCG and gemcitabine. AsPC-1 and PANC-1 cells were treated with EGCG (20 µM) or gemcitabine (0.5 µM) for 48 h. The expression of VEGF, c-Myc, survivin and cyclin D1was was measured by qRT-PCR. Data represent mean ± SD. * = significantly different from respective controls, P<0.05.

Gemcitabine has emerged as a popular chemotherapeutic agent in the treatment of advanced and metastatic pancreatic cancer, and the benefit of this single-agent is small but significant in the improvement of median overall survival [19]. To test the effects of gemcitabine with EGCG on cell viability, pancreatic cancer cells were treated with gemcitabine (0.5 µM) with or without increasing concentrations of EGCG (0–60 µM) for 72 hours. As shown in Fig. 5A, cell viability was inhibited by EGCG and gemcitabine alone, and the inhibitory effects of gemcitabine on cell viability were further enhanced by EGCG in these two cell lines (Fig. 6A).

We next examined the interactive effects of EGCG with gemcitabine on apoptosis in both AsPC-1 and PANC-1 cell lines (Fig. 6B). EGCG induced apoptosis in both the cell lines in a dose-dependent manner. Similarly, gemcitabine induced apoptosis in both AsPC-1 and PANC-1 cells. All the doses of EGCG further enhanced the effects of gemcitabine on apoptosis. These data suggest that EGCG can be combined with gemcitabine to treat pancreatic cancer patients.

We next examined the effects of EGCG and gemcitabine on the expression of c-Myc and cyclin D1 in AsPC-1 and PANC-1 cells (Fig. 6C). EGCG and gemcitabine alone inhibited the expression of VEGF, c-Myc, survivin and cyclin D1was in both the cell lines. The combination of EGCG and gemcitabine had additive effects on these target genes. These data suggest that EGCG can enhance the therapeutic potential of gemcitabine in pancreatic cancer cells by inhibiting STAT3.

### JAK3 inhibitor CP690550 inhibits cell viability in pancreatic cancer cells

**Figure 7:**
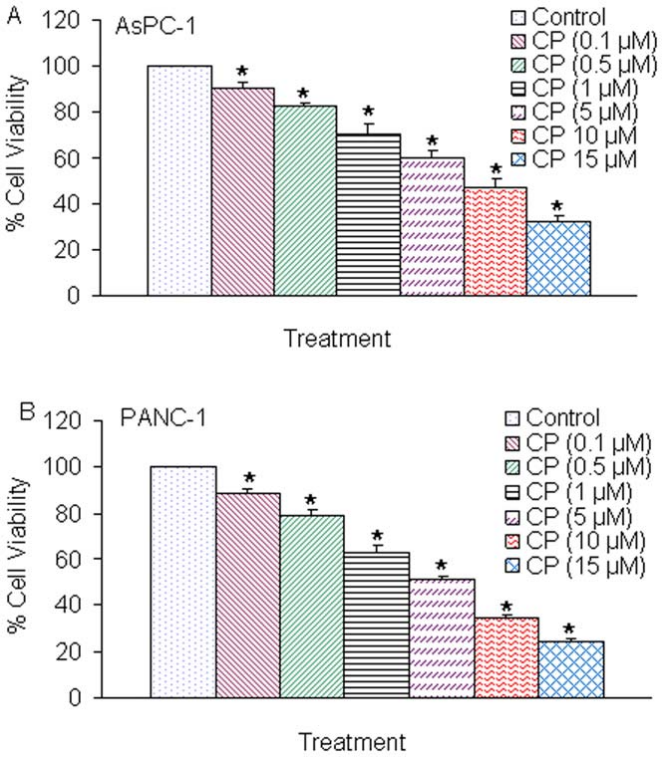
CP690550 inhibits cell viability of AsPC-1 and PANC-1 cells. (A), AsPC-1 cells were seeded in 96 well plates at 4×10^4^ cells per well and treated with CP690550 (0–15 µM) for 72 h. Cell viability was measured by XTT assay. Data represent mean ± SD. * = significantly different from control, P<0.05. (B), PANC-1 cells were seeded in 96 well plates at 4×10^4^ cells per well and treated with CP690550 (0–15 µM) for 72 h. Cell viability was measured by XTT assay. Data represent mean ± SD. * = significantly different from control, P<0.05.

CP690550 is a novel JAK3 inhibitor and expected to target JAK3, which is expressed generally only in immune cells and is only bound by gamma-chain-bearing cytokine receptors involved in the JAK/STAT signaling pathway [37]. We next examined the effects of CP690550 on cell viability in AsPC-1 and PANC-1 cells (Fig. 7). CP690550 inhibited cell viability in both the cell lines in a dose-dependent manner. These data suggest that CP690550 can be a potential anticancer drug for the treatment of pancreatic cancer.

### CP690550 synergizes with EGCG to inhibit cell viability in pancreatic cancer cells

**Figure 8:**
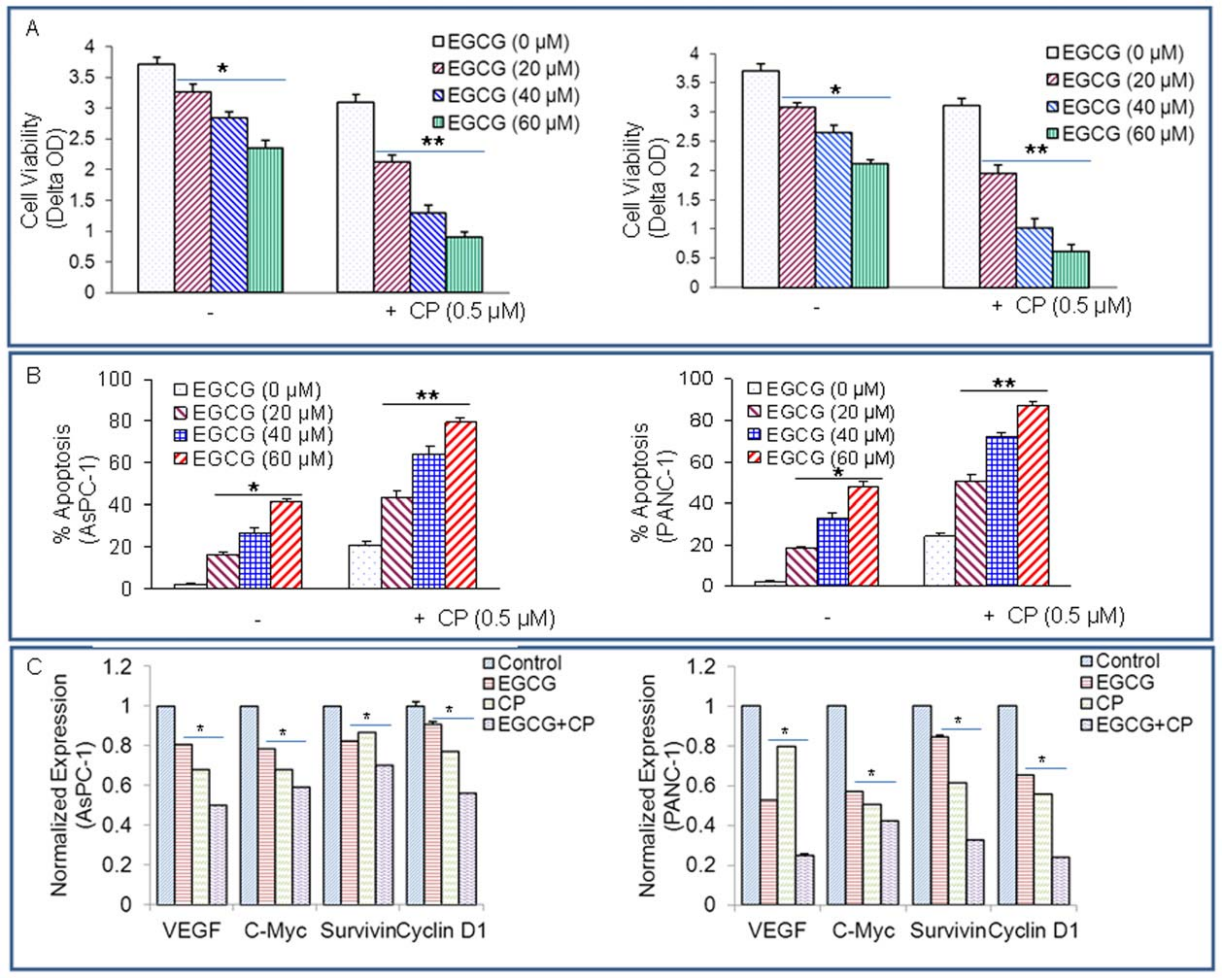
EGCG and CP690550 inhibit cell viability and STAT3 target genes. (A), AsPC-1 and PANC-1 cells were treated with EGCG (0, 20, 40, 60 µM) with or without CP690550 (0.5 µM) for 72 h. Cell viability was measured by XTT assay. Data represent mean ± SD. * or ** = significantly different from respective controls, P<0.05. (B), AsPC-1 and PANC-1 cells were treated with EGCG (0, 20, 40, 60 µM) with or without CP690550 (0.5 µM) for 72 h. Apoptosis was measured by TUNEL assay. Data represent mean ± SD. * or ** = significantly different from respective controls, P<0.05. (C), Inhibition of STAT3 target genes by EGCG and CP690550. AsPC-1 and PANC-1 cells were untreated or treated with EGCG (20 µM) or CP690550 (0.5 µM) for 48 h. The expression of VEGF, c-Myc, survivin and cyclin D1 was measured by qRT-PCR. Data represent mean ± SD. * = significantly different from respective controls, P<0.05.

Since CP690550 induced apoptosis in pancreatic cancer cells, we next sought to examine the interactive effects of CP690550 and EGCG on cell viability and apoptosis of pancreatic cancer cells (Fig. 8). To test the effects of CP690550 with EGCG on cell viability and apoptosis, pancreatic cancer cells were treated with CP690550 (0.5 µM) and increasing concentrations of EGCG (0–60 µM) for 72 hours. As shown in Fig. 8A and B, EGCG and CP690550 inhibited cell viability and induced apoptosis in both AsPC-1 and PANC-1 cell lines. Interestingly, EGCG further enhanced the effects of CP690550 on cell viability and apoptosis in both the cell lines. These data suggest that CP690550 can be combined with EGCG to target pancreatic cancer cells.

Since EGCG and CP690550 inhibited cell viability and induced apoptosis, we next sought to examine their effects on STAT3 target genes. EGCG and CP690550 inhibited the expression VEGF, c-Myc, survivin and cyclin D1was in both the cell lines (Fig. 8C). The combination of EGCG and CP690550 had additive effects on these target genes. These data suggest that EGCG can enhance the therapeutic potential of CP690550 in pancreatic cancer cells by inhibiting STAT3.

## Discussion

Pancreatic cancer belongs to the group of extremely aggressive human cancers; conventional treatments have little impact. In 2010, it accounts for only 3% of new cancer cases in the United States, and the fourth leading cause of cancer death. Only 6 percent of patients will survive 5 years after diagnosis [38]. Various factors, which include its aggressive nature, lack of early screening, absence of therapeutic targets, and lack of effective treatments, make the pancreatic cancer become one of the most difficult cancers to treat. In recent years, the clinicians and cancer scientists have made some significant advances into the management of the disease, especially discovery and development of targeted therapeutics [39].

Many oncogenic molecular pathways including EGF/EGFR, Ras-Raf-MEK, PI3K/Akt, JAK/STAT, p16INK4A/retinoblastoma, Smad4/TGF-β, and hedgehog signaling pathways, have been reported to be involved in the pathogenesis of pancreatic cancer [40], [41], [42], [43], [44], [45]. Among them, STAT3 is thought by many researchers as a suitable therapeutic target for drug discovery because constitutive activation of STAT3 alone is sufficient to induce the relevant disease, the inhibition of STAT3 signaling could suppress and reverse the development of relevant disease, and the molecular mechanism of tumorigenesis caused by STAT3 pathway has been well defined.

Chemoprevention was first defined by Sporn in 1976 and refers to the use of natural or synthetic agents to reverse, suppress or prevent carcinogenic progression [46]. It has been proved as a rationale and promising strategy by several recent epidemiological studies in preventing cancer in high-risk populations. Because natural compounds are generally cheaper and safer than synthetic ones, there is growing interest in the possible therapeutic potential of natural products against cancer. Many epidemiological, preclinical, and clinical studies have demonstrated the cancer-preventive effects of green tea [47], [48], [49], [50]. The chemotherapeutic and chemopreventive effects of green tea have been attributed to the polyphenol components, especially EGCG, which is the most abundant polyphenol in green tea and accounts for more than 40% of the total polyphenol mixture [51]. In the recent few decades, it was under intensive investigation by using animal models of carcinogenesis and cultured tumor cell lines [29], [52]. EGCG has demonstrated remarkable chemopreventive and chemotherapeutic potential against various types of cancers, e.g. skin, lung, breast, colon, prostate, stomach, and pancreas, by modulating the intracellular signaling network [30], [51], [52], [53].

In the present study, we provide strong evidence that EGCG can inhibit cell viability and induce apoptosis of pancreatic cancer cells. First, we found that the expression and activation of STAT3 were inhibited by EGCG, while the induction of caspase-3 activity and PARP cleavage were enhanced. Moreover, this compound also inhibited the invasion and migration of pancreatic cancer cells, which has been reported to be implicated with STAT3 [54]. These results demonstrate that EGCG has a marked anti-cancer effect on pancreatic cancer at least in part by the inhibition of STAT3 signaling pathway. Second, we found that the STAT3 shRNA can alone reduce cell motility and viability of cancer cells. Furthermore, STAT3 shRNA can enhance the inhibitory effects of EGCG on cell motility and viability in pancreatic cancer cells, suggesting that EGCG can influence some other gene/pathway besides STAT3.

We also found that EGCG could suppress the expression of STAT3-downstream genes, which include the angiogenic protein VEGF, cell proliferative Cyclin D1, oncogenic transcription factor c-Myc, and several anti-apoptotic proteins, including survivin and Bcl-X_L_. Some genes are prominent targets for both NF-κB and STAT3, such as Cyclin D1, Bcl-X_L_ and c-Myc, while survivin is STAT3-dependant. VEGF is also controlled by STAT3 and might be indirectly regulated by NF-κB via HIF-1α [55], [56], [57], [58]. The EGCG-medicated inhibition of Cyclin D1, VEGF, and BclX_L_ transcription is consistent with previous reports [59], [60], [61], which might result from the suppression of EGCG against both the NF-κB and STAT3 pathways [62]. Similarly, the EGCG inhibition on Wnt signaling and PI3K/Akt could also contribute to the down-regulation of cMyc and survivin, respectively [63], [64].

Pancreatic cancer is poorly treated by conventional chemotherapies including gemcitabine due to the profound chemoresistance through widely expressed HMGA1 [65]. CP690550 (Tasocitinib), an orally active immunosuppressant, is being developed by Pfizer for the treatment of inflammatory bowel disease, dry eyes, rheumatoid arthritis, ankylosing spondylitis, psoriasis, psoriatic arthritis, and for the prevention of transplant rejection [21], [66], [67], [68], [69], [70], [71]. CP690550 specifically inhibits JAK3, which has a pivotal role in cytokine signal transduction that governs lymphocyte survival, proliferation, differentiation, and apoptosis. Recent studies have demonstrated the anticancer activity of CP690550 in various cancers [72]. In this study, we found that gemcitabine, CP690550 and EGCG alone inhibited cell viability, induced apoptosis and attenuated STAT3-regulated gene transcription in AsPC-1 and PANC-1 cells. EGCG further enhanced the effects of gemcitabine or CP690550 on cell viability, apoptosis and on the expression of STAT3-target genes. Our results provide a new application method, in which the use of EGCG can enhance the therapeutic effects of anticancer drugs while possibly reducing their side effects.

In conclusion, our findings provide unprecedented insights into the STAT3 signaling pathway by which EGCG inhibits viability, invasion and migration, and induces apoptosis in pancreatic cancer cells. Inhibition of STAT3 by shRNA could suppress viability of cancer cells, and down-regulate the STAT3-target genes. Most importantly, EGCG further enhanced the therapeutic potential of gemcitabine and CP690550 against pancreatic cancer.
